# Fucoidan: An Update on Function, Role in Human Health and Applications

**DOI:** 10.1111/cbdd.70310

**Published:** 2026-05-09

**Authors:** Sharon Critelli, Marilena Celano

**Affiliations:** ^1^ Department of Health Sciences University “Magna Graecia” of Catanzaro Catanzaro Italy

**Keywords:** adjuvant, anticancer, antioxidant, autophagy, drug delivery system, epigenetic, exosomes, fucoidan

## Abstract

Fucoidan is a polysaccharide bioactive compound, mainly from brown algae, characterized by multiple biological activities, including anti‐inflammatory, anticoagulant, anticancer, antioxidant, antiviral, and cardioprotective properties. In addition, growing scientific evidence supports its epigenetic potential and ability to modulate autophagic mechanisms, two crucial aspects for the treatment and prevention of chronic diseases, such as cancer, inflammation, and aging. For this reason, Fucoidan is a promising candidate for a wide range of applications, both as a bioactive component of drug delivery systems in order to improve their stability, bioavailability, and potentially reduce their side effects, and as an adjuvant in drug therapies. The aim of this review is to provide an update on the correlation between properties and distinctive chemical characteristics, to underline an interesting emerging role in epigenetics and autophagy and to be a new key point for preclinical and clinical studies, in order to better understand their properties and possible new therapeutic and biomedical applications.

## Graphical abstract

Fucoidan is a promising bioactive compound with anti‐inflammatory, anticoagulant, anticancer, antioxidant, antiviral, and cardioprotective properties. In addition, thanks to its epigenetic potential, ability to modulate autophagic mechanisms and possibility adjuvant agent or bioactive component of the drug delivery systems, fucoidan is a promising candidate for several nutraceutical and pharmaceutical applications.

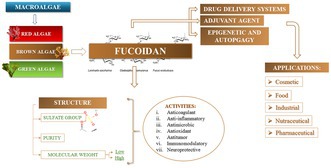

## 1 Introduction

In the last decade, biologists have identified over 10,000 bioactive substances derived from algae (Bošnjaković and Sinaga 2020; Lisha et al. 2022; Trubetskaya et al. 2024), such as polysaccharides, proteins, lipids, polyphenols, carotenoids, pigments, vitamins, sterols and enzymes (Chandini and Ponesakki 2008; Garcia‐Vaquero et al. 2017). These molecules give algae different functional properties and biological effects (Babich et al. 2022), offering a wide range of benefits for human health (Sharma et al. 2023).

Algae can be divided into two main categories: macroalgae, multicellular benthic organisms, commonly known as seaweeds, and microalgae, single‐celled eukaryotic organisms (Bertrand et al. 2016; Frazzini et al. 2022; Mapelli‐Brahm et al. 2023; Ruiz Martínez et al. 2024; Trubetskaya et al. 2024).

In particular, macroalgae, based on the type of pigments, can be classified into three main groups: red algae (*Rhodophyta*), brown algae (*Phaeophyta*), and green algae (*Chlorophyta*) and are particularly valued for their content of bioactive compounds (Table 1) (Bleakley and Hayes 2017; Frazzini and Rossi 2025).

**TABLE 1:** Classification of macroalgae and their functional compounds.

Compounds	Macroalgae		
	Red algae	Green algae	Brown algae
Carotenoids	2‐Bromophenol	Siphonaxanthin	Fucoxanthin Fucoxanthinol Siphonaxanthin Xanthophylls
Phenols	—	—	Phloroglucinol Phlorotannins
Polysaccharides	Carrageenan Porphyran	Agarose, Ulvan Rhamnan sulfate	Alginate Fucoidan Laminarin
Quinones	—	—	Hydroquinone Quinone
Chlorophylls	—	Chlorophyll a Chlorophyll b	—
Sterols	Phytosterol	Fucosterol	Fucosterol Phytosterol
Terpenoids	Monoterpene	—	Diterpenoid Triterpenoid

Brown algae, one of the main groups of macroalgae, synthesize a high number of carotenoids, responsible for their characteristic greenish‐brown coloration (Kergosien et al. 2023; Reboleira et al. 2019) and their cell walls are made up of an amorphous matrix of polysaccharides such as fucoidan, alginic acid, and laminarin, which ensure their flexibility and structural integrity (Cunha and Grenha 2016).

Among these polysaccharides, fucoidan stands out for its peculiar biological properties and its promising applications in the pharmaceutical, nutraceutical, food, and cosmetic fields.

**FIGURE 1:**
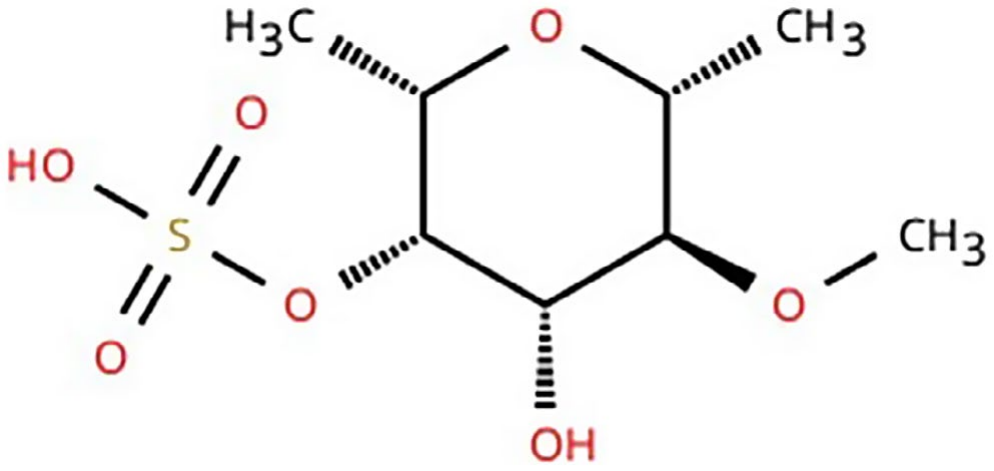
Chemical structure Fucoidan. IUPAC nomenclature [(2S,3S,4S,5S,6R)‐4‐hydroxy‐5‐methoxy‐2,6‐dimethyloxan‐3‐yl] hydrogen sulfate.

Fucoidan, also called fucan or fucosane, is a water‐soluble heteropolysaccharide, commonly extracted from brown algae belonging to several species, including *Ecklonia cava, Ascophyllum nodosum, Cladosiphon okamuranus, Undaria pinnatifida, Saccharina longicruris, Saccharina latissima, Sargassum polycystum, Laminaria japonica, Fucus vesiculosus*, and *Fucus serratus* (Bilan et al. 2010; Bilan et al. 2006; Bilan et al. 2013; Chevolot et al. 2001; Elizondo‐Gonzalez et al. 2012; Rioux et al. 2010; Wijesinghe and Jeon 2012; Zhao et al. 2018). Fucoidans are characterized by an α‐fucopyranose backbone linked to monosaccharides (glucose, mannose, galactose), sulfate groups, and acetyl groups, and it is characterized by a highly variable molecular weight (MW) ranging from 100,000 to 5,200,000 Da (Li et al. 2008; Suprunchuk 2019; Wang et al. 2020; Yu et al. 2021) (Figure 1).

Although the distribution of sugar residues and acetyl groups does not follow a standard pattern, the arrangement of glycosidic bonds appears conserved, with species‐dependent variations (Cunha and Grenha 2016; George and Shrivastav 2023). Furthermore, the presence and position of sulfate groups, which confer a negative charge to the polymer, are essential for its activity (George and Shrivastav 2023). Sulfation occurs selectively at the C‐2 and/or C‐4 positions of the fucose units, while substitution at the C‐3 position is chemically less frequent, and it is also species‐dependent (Ale et al. 2011; George and Shrivastav 2023; Hahn et al. 2012).

**FIGURE 2:**
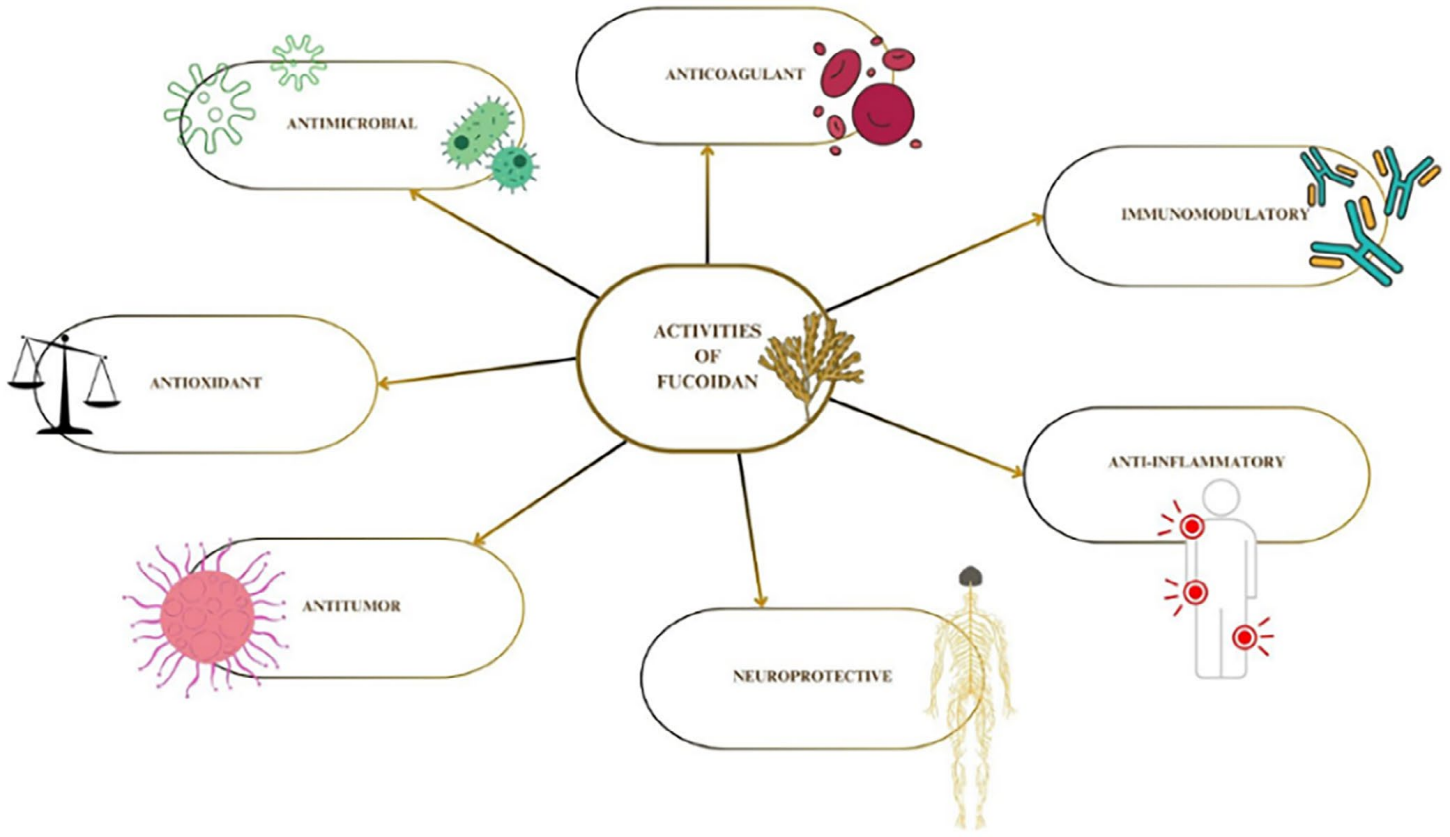
Biological activities of fucoidan.

Fucoidan thanks to its heterogeneous composition and given its numerous biological properties (anti‐inflammatory, anticoagulants, anticancer, antioxidants, antivirals, and cardioprotective) (Figure 2) has attracted the attention of researchers (Guerra‐Rivas et al. 2011; Huwait et al. 2022; Lee and Lee 2006; Parvez et al. 2019; Raguz and Yagüe 2008; Rioux et al. 2007; Ustyuzhanina et al. 2013; Yang et al. 2008; Zhao et al. 2016). In particular, the role of fucoidan as a modulator of key cellular processes, such as epigenetic regulation and autophagy, has recently aroused greater interest.

The biological properties of fucoidan are strongly influenced by molecular weight, chemical structure, algal species of origin, harvesting period, geographical location of the algae, and the extraction method adopted (Zayed et al. 2020; Zayed et al. 2016).

The use of high temperatures can cause the breakage of molecular bonds leading to the fragmentation of the structure and/or the use of chemical agents can introduce new functional groups, altering and modifying, consequently, the polysaccharide composition (Silva et al. 2022). In addition, the high number of branching points of the molecular skeleton gives it an additional level of structural complexity (Silchenko et al. 2017; Zayed et al. 2020).

The present review aims to analyze the biological effects of fucoidan, paying attention to the correlation of these activities with the structural parameters of the polysaccharide, such as molecular weight (MW), degree of purity, concentration, and presence of specific functional groups, in particular sulfate groups. The role of fucoidan in the modulation of autophagy and epigenetic regulation will also be described, highlighting its potential therapeutic implications in different pathological contexts. Finally, the main areas of biomedical application of fucoidan will be examined, with a specific focus on its use as a drug delivery system and adjuvant in chemotherapy.

## 2 Molecular Weight, Sulfate Group, Purity and Concentration of Fucoidan

Numerous studies have shown that the bioactivity of fucoidan is closely related and strongly influenced by specific structural parameters. The molecular weight of fucoidan is a determining factor that can influence its biological activity.

Low molecular weight (LMW) fucoidan exhibits higher absorption, higher bioavailability, and more marked biological effects (higher antioxidant, anti‐inflammatory, antitumor, neuroprotective, cardioprotective, and antiviral), compared to high molecular weight (HMW) counterparts (Zhang et al. 2010).

In the case of native fucoidan extracted from *Saccharina japonica* (136,000 Da), the peak time (t_peak_) is 2.5 h, while its LMW counterpart (9500 Da) shows a faster absorption, with a shorter peak time and a significant increase in the area under the curve (AUC) (Tan et al. 2023; Zhao et al. 2016). Similar results were also obtained with fucoidan extracted from *Laminaria japonica*, administered orally both in the native form (90,400 Da) and in the respective LMW form (5500 Da) (Sun et al. 2024; Zhao et al. 2016). In this case, although the two forms showed similar values of t‐peak, half‐life and AUC, the maximum plasma concentration (C_max_) was significantly higher in LMW fucoidan (Sun et al. 2024; Zhao et al. 2016). Other pharmacokinetic parameters confirm a more efficient systemic distribution for LMW fractions (8000 Da), which have a clearance of 0.88 ± 0.46 mL/min and a volume of distribution of 57.6 ± 27.9 mL (Deux et al. 2002). After oral administration, the tissue distribution of LMW fucoidan partly reflects that of native fucoidan (Pozharitskaya et al. 2018), but with a predominantly renal localization (Sun et al. 2024; Tan et al. 2023; Zhao et al. 2016).

From an anti‐inflammatory perspective, LMW fucoidan has shown increased efficacy in several in vitro and in vivo models, acting mainly through three mechanisms: the reduction of oxidative stress through the activation of the Nrf2/ARE pathway, the modulation of the gut microbiota and the inhibition of the expression of pro‐inflammatory mediators mediated by interference with NF‐κB signaling pathways, PI3K, TGF‐β/Smad and MAPK (Luo and Liu 2025). Potential molecular targets include Toll‐like receptors type 4 (TLR‐4), key receptors upstream of the NF‐κB cascade, and P‐selectin, whose ligand interaction is blocked by LMW fucoidan (Bachelet et al. 2009).

In mice, administration of LMW fucoidan led to a significant reduction in the levels of reactive oxygen species (ROS) and malondialdehyde (MDA), an increase in reduced glutathione (GSH), accompanied by an enhancement of the activity of key enzymes involved in the antioxidant response, such as glutathione peroxidase (GSH‐Px), superoxide dismutase (SOD), catalase (CAT), heme oxygenase‐1 (HO‐1), and NAD(P)H: quinone oxidoreductase 1 (NQO1) (Deng et al. 2022; Deng et al. 2021; Dong et al. 2022; Guan et al. 2020; Wang, Zhang, Jin, et al. 2011; Wang, Zhang, Zhang, et al. 2011; Zheng et al. 2018).

Particularly relevant are the results obtained with LMW fucoids extracted from *Laminaria japonica*, which also exerted significantly greater efficacy in inhibiting the oxidation of low‐density lipoproteins (LDL) compared to high‐molecular‐weight fractions, suggesting a potential protective role against atherosclerosis and other diseases related to oxidative stress (Zhao et al. 2011). LMW fucoids also possess a greater capacity to inhibit cell proliferation and induce apoptosis in several tumor cell lines (breast, stomach, liver cancer) through calcium‐dependent mitochondrial mechanisms and caspase activation (Choi and Kim 2013; Lu et al. 2018; Zhang et al. 2013). They can also induce apoptosis through a p53‐independent mechanism, as seen in colon cancer cells (Park et al. 2017).

In addition, the antitumor activity, attributed to the ability of the polysaccharide to interact with TLR4 and to modulate several cell signaling pathways, including those mediated by Bone Morphogenetic Protein Receptor (BMPR), Transforming Growth Factor‐beta Receptor (TGFR) and Epidermal Growth Factor Receptor (EGFR) (Park et al. 2017), is also influenced by parameters such as concentration, purity and MW of fucoidan (Cumashi et al. 2007).

Recently, a LMW (< 667 kDa) form, called oligo‐fucoidan (OF), extracted from *Laminaria japonica*, has promoted differentiation in malignant glioma (MG) cells and downregulation of the expression of DNA methyltransferases (DNMT) 1, 3A and 3B (Liao et al. 2019), suggesting a potential epigenetic mechanism linked to DNA demethylation (Liao et al. 2019; Turcan et al. 2013). OF determined the induction of p21 in U87MG cells and the demethylation of its promoter (Liao et al. 2019). Further evidence indicates that OF reduces DNMT3B expression through the induction of miR‐29b (Yan et al. 2015), a microRNA known to directly target DNMT3A and 3B, and indirectly DNMT1 via suppression of the transcription factor Sp1 (Garzon et al. 2009).

The blood–brain barrier (BBB) is permeable to a molecular weight estimated to be around 400 Da, so OF could potentially cross the BBB more efficiently than native fucoidan, which features an MW of more than 700,000 Da (Pardridge 2012; Pozharitskaya et al. 2018). This aspect is particularly relevant in the treatment of glioblastoma multiforme, in which the permeability of the blood–brain barrier is frequently compromised due to the loss of tight junction integrity, induced by VEGF (Wen et al. 2017), and structural alterations of the endothelium (Takeuchi et al. 2004). OF, due to its potential to exceed BBB, its low toxicity, and synergism of action with approved epigenetic agents, is a promising molecule for the therapy of malignant glioma (Liao et al. 2019).

MW also strongly influences anticoagulant activity. Significant anticoagulant activity was observed in fucoidans with MW between 50 and 100,000 Da, while fractions with a MW greater than 850,000 Da did not appear to have significant anticoagulant effects. For example, fucoidan at LMW (7600 Da) isolated from *Laminaria japonica* showed superior antithrombotic activity compared to high molecular weight fucoidan (Zhao et al. 2016).

Fucoidans with a molecular weight between 10,000 Da and 300,000 Da have also been shown to be particularly effective in preventing blood clotting (Yang et al. 2008).

In cardiac aging in mice, characterized by reduction in autophagy and autophagic flow resulting in the accumulation of misfolded proteins and dysfunctional organelles, fucoidan resulted in a protective effect (Chang et al. 2019; Mialet‐Perez and Vindis 2017). In rat embryonic myocardial cells, H9C2, treatment with fucoidan (50 μg/mL) resulted in increased expression of Beclin‐1 and LC3‐II, inducing the JAK2‐STAT3 signaling pathway (Zhang et al. 2020). In addition, in the same cells, LMW derivatives of fucoidan (alginate oligosaccharide, easily absorbed) delayed H_2_O_2_‐induced senescence, inducing the mRNA expressions of LC3‐II/LC3‐I and reducing p62 (Feng et al. 2024; Gupta et al. 2020; Tocaciu et al. 2018).

Fucoidan has also shown promising neuroprotective effects, promoting neuronal health and improving cognitive function in several neurodegenerative diseases such as Alzheimer's and Parkinson's (Wei et al. 2017; Zhang et al. 2018). Neuroprotective activity has been associated with its ability to reduce oxidative stress, modulate apoptotic processes, and regulate the cholinergic system (Gao et al. 2012; Ignacimuthu 2020; Wei et al. 2017; Zhang et al. 2018).

In addition, increases in the levels of key neurotransmitters, such as dopamine, glutamate, GABA, tryptamine, and serotonin, have been observed (Ignacimuthu 2020). In particular, LMW fucoidan has been shown to be particularly effective in supporting memory functions, regulating brain metabolism, and increasing levels of neurotrophic factors essential for neuronal plasticity (Zhang, Wu, et al. 2022).

Several studies have also documented the antiviral efficacy of fucoids extracted from different species of algae (Queiroz et al. 2008). In this context, fucoidans extracted from the genus *Fucus* spp. showed higher virucidal activity than other species such as *Sargassum scroederi* and *Digenea mertensii* (Queiroz et al. 2008). Furthermore, LMW derivatives of fucoidan have recently been formulated, some of which have demonstrated the ability to bind the spike protein of the SARS‐CoV‐2 virus (Koike et al. 2021).

Among these, fucoidan 2,3‐O‐sulfate has been identified as the compound with the greatest inhibitory activity, surpassing in terms of efficacy even Fondaparinux, currently used in therapy (Koike et al. 2021). However, despite its antiviral efficacy in vitro, its poor bioavailability in vivo still limits its use as an antiviral agent (Lüscher‐Mattii 2000).

The biological efficacy of fucoidan is also strongly influenced by the total sulfate content, the fucose/sulfate ratio, and the position of the sulfate groups along the polysaccharide chain. It has been observed that a high degree of sulfation is often associated with an increase in bioactivity, although this relationship is not always linear. At the same time, chemical modifications such as desulfation or deacetylation can reduce or alter its biological properties (Ferreira et al. 2015; Khil'chenko et al. 2011).

Several studies also document the antioxidant activity of fucoidan in vivo (Deng et al. 2022; Deng et al. 2021; Dong et al. 2022; Guan et al. 2020; Wang, Zhang, Jin, et al. 2011; Zheng et al. 2018), showing that it acts primarily as a secondary antioxidant (Koh et al. 2019).

Several in vitro and in vivo studies have shown that antioxidant activity also depends on concentration, particularly at low doses (7.1 ± 0.01 μg/mL); fucoidan showed a scavenging activity of 50% on the free radicals generated and, at higher concentrations (73.3 μg/mL), a scavenging activity of more than 90% on the superoxide radical (Wang et al. 2009).

The presence of sulfate groups, as well as the ratio of fucose to sulfate content, directly affects the ability of fucoidan to neutralize reactive oxygen species, particularly hydroxyl radicals (Wang et al. 2008; Wang et al. 2010). The specific location of sulfate ester groups plays a crucial role in increasing its scavenging activity (Zhao et al. 2011).

The degree of purity and concentration of fucoidan are further fundamental parameters in the modulation of its biological activities.

Purified fucoidans extracted from *Turbinaria decurrens* showed remarkable anti‐inflammatory activity, mediated by the inhibition of pro‐inflammatory enzymes and key molecular mediators such as nitric oxide synthase (iNOS), cyclooxygenase‐2 (COX‐2), nitric oxide (NO), pro‐inflammatory cytokines, IL 1β, and matrix metallopeptidases 9 (MMP‐9) (Dinesh et al. 2016; Jayawardena et al. 2020; Manikandan et al. 2020; Wang et al. 2020).

Fucoidans with a high sulfate group content and a higher amount of fucose also showed increased anti‐inflammatory activity (Jayawardena et al. 2020; Sanjeewa et al. 2018). A study of Kirindage et al. has documented how fucoidan extracted from *Saccharina japonica*, containing 29.82% fucose and 15.82% sulfate, reduces the activity of pro‐inflammatory cytokines such as tumor necrosis factor alpha (TNF‐α) and interferon gamma (IFN‐γ) and interleukins (IL) such as IL‐6, IL‐8, IL‐13, IL‐25 and IL‐33 (Kirindage et al. 2024). Moreover, several studies also indicate a clear dose–response relationship, in which the anti‐inflammatory capacity is influenced not only by the chemical composition (e.g., fucose content and sulfate groups) but also by the concentration administered (Obluchinskaya et al. 2022).

In mouse models, the dose of 250 mg/kg fucoidan determined significantly marked anti‐inflammatory effects compared to lower doses (10 and 50 mg/kg), although not superior to the positive control (diclofenac sodium) (Manggau et al. 2022).

Furthermore, the antitumor activity is significantly influenced by the presence of sulfate groups in the structure, the degree of purity and the concentration (Ermakova et al. 2011).

In particular, fucoidans with a high degree of sulfation show greater antitumor activity than those with a low sulfate group content (Ermakova et al. 2011). In one study, a sulfate derivative (2,4‐O‐sulfate) of LMW fucoidan showed greater antitumor efficacy than native fucoidan against triple‐negative breast cancer cells (Zueva et al. 2023). Similarly, a synthetic fucoidan derivative obtained from *Fucus vesiculosus* , having a 3,4‐O‐sulfate fucosate structure, showed significant antiproliferative activity against MCF‐7 (breast cancer) and HeLa (cervical cancer) cell lines, with a dose‐dependent effect (Kasai et al. 2015). Native fucoidan extracted from *Saccharina dentigera*, at a concentration of 200 μg/mL, exerted a reduction of 87%, 31% and 22% in cell proliferation, on human duodenal adenocarcinoma cells (HuTu 80), malignant melanoma (RPMI‐7951) and colorectal cancer cells (HCT‐116), respectively (Usoltseva et al. 2022). Dose‐dependent induction of apoptosis has also been observed in breast cancer (MCF‐7), hepatocellular carcinoma (HepG2), lung cancer (NCI‐H460), and HeLa tumor cell lines (Hoang et al. 2022; Usoltseva et al. 2022).

In gastric cancer, fucoidan treatment on human gastric adenocarcinoma (AGS) cells, at a dose of 300 μg/mL, significantly increased autophagosome formation, compared to control groups, and the conversion of LC3‐I to LC3‐II was upregulated, as well as the expression of Beclin‐1 (Park et al. 2011).

Similar results were observed in multiple myeloma, where treatment with 200 μg/mL of fucoidan of U266 cells increased the expression of LC3‐II/LC3‐1 and the Beclin‐1 protein in a concentration‐dependent manner, while p62 levels were reduced (Luo et al. 2017). In addition, it led to a dose‐dependent reduction in MMP‐9 secretion and chemokine receptor type 4 (CXCR4) expression, biomarkers of myeloma cell migration and invasion (Luo et al. 2017).

In myeloma cells, fucoidan also reduced AKT phosphorylation, inhibiting downstream m‐TOR phosphorylation, demonstrating the potential role as a modulator of autophagy through downregulation of the AKT/m‐TOR signaling pathway (Zhang, Xue, et al. 2022). In hepatocellular carcinoma, both native and partially hydrolyzed fucoidan isolated from *Fucus vesiculosus* inhibited the proliferation of HepG2 cells at a concentration of 180 μg/mL, compared to normal hepatocytes (Chang liver cells), and induced apoptosis and autophagy, with an increase in acidic vesicular organelles (Zhurishkina et al. 2020).

The presence and localization of sulfate and acetyl groups on fucoidan have been shown to be important factors also in determining its immunomodulatory activity (Choi et al. 2005).

Immunomodulatory properties can enhance and/or suppress the activity of the immune system, and this is advantageous as a well‐regulated immune response can help limit tumor proliferation and improve the effectiveness of drug therapies (Wang et al. 2019). In particular, fucoidan interacting with TLRs present on dendritic cells, macrophages, and other monocytes can stimulate the release of pro‐inflammatory factors, cytokines and chemokines, thus contributing to the activation of an effective immune response (Akira et al. 2006; Zhang et al. 2015). Moreover, fucoidans are characterized by a low sulfate content but, with high structural heterogeneity, still show powerful immunomodulatory activity.

However, it is important to note that while some algal polysaccharides stimulate anti‐cancer immune responses, others may cause side effects such as excessive inflammation or immune suppression, resulting in an increased risk of autoimmune reactions or infections (Murphy et al. 2023).

The antithrombotic and anticoagulant activities of fucoidan are also strongly influenced by the degree of sulfation and fucoids with a high sulfate group content have been shown to be more effective than those with lower sulfate contents (Chevolot et al. 2001; Wang, Zhang, Zhang, et al. 2011; Wang et al. 2010).

In addition, fucoidan has been recognized as a potent broad‐spectrum antiviral agent, characterized by low toxicity and low onset of resistance (Queiroz et al. 2008).

Several studies in vitro and in vivo have demonstrated that the interaction between sulfate groups of fucoidan and positively charged glycoproteins present on the surface of the viral envelope prevents the viral infection (Jiao et al. 2012; Tan et al. 2022). Fucoidan has shown significant antiviral activity against several viruses such as *human immunodeficiency virus* (*HIV‐1*) by blocking the early stages of viral entry into cells (Damonte et al. 2004; Iqbal et al. 2000; Mandal et al. 2007; Thuy et al. 2015), *herpes simplex virus* (*HSV*), *human cytomegalovirus*, *influenza virus*, and *bovine viral diarrhea virus* (Dinesh et al. 2016; Feldman et al. 1999; Iqbal et al. 2000; Jiao et al. 2012; Lee et al. 2004; Mandal et al. 2007; Ponce et al. 2003; Witvrouw and De Clercq 1997). At the same time, it also showed antifungal activity, closely related to the presence of sulfate groups and to the structure of the sugar skeleton (Phull, Ali, et al. 2017; Phull, Majid, et al. 2017). In particular, fucoidan extracted from *U. pinnatifida* has shown fungicidal activity against pathogenic strains such as *Aspergillus flavus* (FCBP‐0064), *Aspergillus fumigatus* (FCBP‐66), and *Mucor* spp (FCBP‐0300), and further research has documented its fungicidal activity against *Candida albicans* , *Candida glabrata*, and *Candida parapsilosis* , through the inhibition of DNA and protein synthesis and damage to cell membranes (Alghuthaymi et al. 2021).

## 3 Fucoidan: Epigenetic Modulation, Gut‐Microbiota Axis and Skin Aging

Epigenetic regulation plays a crucial role in cell differentiation, embryonic development and in the self‐renewal of cancer stem cells, therefore is becoming a strategic target for the development of new differentiated therapeutic approaches in solid tumors (Kawamura et al. 2018). The use of compounds capable of restoring a physiological epigenetic profile is configured as a promising therapeutic approach. Alongside, the ability of fucoidan to modulate autophagy, a process essential for the maintenance of homeostasis and cell survival, has been documented (Levy et al. 2017). This process is particularly important in tissues with high energy demand, where it supports ATP production and promotes cell survival in conditions of nutritional deficiency (Delbridge et al. 2017; Koleini and Kardami 2017; Li et al. 2016; Maejima et al. 2016). Fucoidan has been shown to modulate this balance by selectively interfering with the major signaling pathways involved (Hsu and Hwang 2019). In this scenario, exosomes derived from mesenchymal stem cells (MSCs) also emerge, showing strong regenerative and immunomodulatory properties thanks to their content in microRNAs (miRNAs), messenger RNA (mRNA) and proteins (Bao and He 2021), whose biological activity has been enhanced by pretreatment with fucoidan (Lou et al. 2023). These exosomes could in turn influence the behavior of recipient cells, also through epigenetic regulation (Bao and He 2021). Fucoidan's anti‐inflammatory properties have sparked growing interest in the treatment of chronic conditions, including ulcerative colitis and rheumatoid arthritis (He et al. 2019; Liu, Zhang, et al. 2022; Phull, Majid, et al. 2017).

Recently, for the treatment of osteoarthritis, an innovative approach has emerged that exploits low molecular weight (LMW) fucoidan to enhance the therapeutic properties of exosomes derived from MSCs.

In particular, the pretreatment of MSCs with fucoidan has made it possible to obtain modified exosomes (F‐MSCs‐Exo) characterized by significantly higher anti‐inflammatory and protective activity than native exosomes (Lou et al. 2023). F‐MSCs‐Exo have been characterized by size, morphology and protein content, showing physical characteristics similar to those of MSCs‐Exo (Lou et al. 2023). However, in vitro and in vivo models of osteoarthritis, they demonstrated an increased ability to suppress IL‐1β‐induced inflammation, reduce macrophage M1 polarization, preserve the extracellular matrix, and induce autophagy in chondrocytes (Lou et al. 2023).

In F‐MSCs‐Exo a high expression of miR‐146b‐5p, known for its ability to suppress the synthesis of inflammatory mediators through the inhibition of TRAF6, determined an upstream regulation of the PI3K/AKT/mTOR pathway, while inhibition of miR‐146b‐5p resulted in a partial loss of the therapeutic effect of F‐MSCs‐Exo (Lou et al. 2023; Gao and Zhang 2021).

However, the possible contribution of other miRNAs present in exosomes has also emerged, such as miR‐23b‐5p, which targets TRAF6 and reduces cell apoptosis, or miR‐615, capable of interfering with the PI3K/AKT pathway by modulating neuronal survival (Cao et al. 2019; Feng et al. 2021). Fucoidan could therefore be an epigenetic modulator capable of enhancing the paracrine activity of MSCs through the release of exosomes enriched in functional miRNAs.

The combination of fucoidan and exosomes therefore represents a safe, stable and promising noncellular approach for clinical application in osteoarthritis, opening up interesting scenarios for other inflammatory and degenerative diseases (Lou et al. 2023).

In diabetic cognitive dysfunction epigenetic alterations, and in particular DNA demethylation, appear to be crucial for the development of the disease (Chen et al. 2024). In the cerebral cortex of diabetic mouse models (MOD), due to DNA demethylation, the levels of 5‐hydroxymethylcytosine and 5‐formylcytosine are reduced (Chen et al. 2024). In this pathological model fucoidan exerts a direct action on DNA, restoring the levels of these markers and mediating the increase in the expression of TET2 (responsible for DNA demethylation), the stability of which is improved by AMPK‐induced phosphorylation (Chen et al. 2024). In mitochondrial metabolism, Fucoidan reduces brain accumulation of succinate, a known TET2 inhibitor, and increases succinate dehydrogenase (SDH) activity (Chen et al. 2024). Removing damaged mitochondria is a crucial process for preserving β cellular function and slowing down the progression of type 1 diabetes mellitus (T1DM) (Mitchell et al. 2013).

Fucoidan demonstrated the ability to induce selective mitochondrial apoptosis in stressed β‐pancreatic cells and to modulate the Bax/Bcl‐2 ratio, caspase 3 and 9 activation and AMPK phosphorylation (Chantree et al. 2021; Li and Chen 2019; Liu, Yang, et al. 2020). In parallel, it stimulates the production of intestinal short‐chain fatty acids (SCFAs), which not only cross the blood–brain barrier but also act as epigenetic modulators and AMPK activators in the cerebral cortex (Chen et al. 2024). In T1DM, fucoidan was distinguished by its marked immunomodulatory activity. In NOD mouse models, it reduced the Th1‐type immune response and increased the population of regulatory T cells (CD4+, CD25+, Foxp3+) (Xue et al. 2019). In treated T1DM mice, an increase in the IL‐4/IFN‐γ ratio in the spleen was also observed, accompanied by a reduction in proinflammatory cytokines (IL‐6, IL‐1β, IFN‐γ, TNF‐α) in the pancreas (Chantree et al. 2021; Liu, Yang, et al. 2020). Due to its recognized anti‐inflammatory, antioxidant, and regenerative properties, it has been successfully used in the formulation of nutraceuticals and incorporated into food products, with the aim of providing additional health benefits (Morton and Agatonovic‐Kustrin 2013; Venkatesan et al. 2022; Zhao et al. 2018).

In particular, supplementation with fucoidan extracted from *A. nodosum* and *L. japonica* resulted in a reduction in opportunistic pathogenic bacteria (such as *Peptococcus*), an increase in beneficial bacteria such as *Lactobacillus* and *Ruminococcaceae*, and a reduction in the inflammatory response and antigenic load (Shang et al. 2016). Other studies confirm its effect in promoting the growth of useful probiotics, such as *Bifidobacterium adolescentis* , *Lactobacillus acidophilus* , and *L. casei* (Liu et al. 2018).

In the last years, fucoidan has also been receiving attention for its potential to counteract the signs of skin aging. Recent studies show that fucoidans extracted from *Undaria pinnatifida* and *Fucus vesiculosus* inhibit enzymes involved in skin aging, such as collagenase and elastase (Fitton et al. 2015), helping to preserve the integrity of elastic fibers and improve skin elasticity (Senni et al. 2006). In addition, fucoidan extracted from *U. pinnatifida*, on the other hand, induces the expression of genes involved in wound healing, contributing to the repair of damaged skin (Fitton et al. 2015). At the cellular level, fucoidan stimulates the upregulation of the SIRT1 protein, improving cellular metabolism and giving the skin a more youthful appearance (Anisha et al. 2022). Considering this evidence, to date, the European Union has approved the use of two fucoidan extracts from *Fucus vesiculosus* and *Undaria pinnatifida* in food supplements and functional products (Lähteenmäki‐Uutela et al. 2021) and is emerging its role in dermocosmetic.

## 4 Fucoidan and Biomedical Application

### 4.1 Adjuvant Agent

A synergism of action between fucoidan and targeted chemotherapy has been demonstrated, particularly in colorectal cancer, with a clinically relevant improvement in the control of disease progression (Tsai et al. 2017).

LMW fucoidans have shown a synergism of action with 5‐fluorouracil in colorectal cancer and oxaliplatin in pancreatic cancer (Deng et al. 2024; Huang et al. 2021).

In triple‐negative breast cancer models, the synergism of action with oligomycin led to an increase in the cytotoxic effect on tumor cells (Zueva et al. 2023). In addition, coadministration of fucoidan with letrozole or tamoxifen in breast cancer patients does not alter the pharmacokinetic parameters of chemotherapy drugs (Tocaciu et al. 2018).

In malignant glioma, OF showed a synergism of action with decitabine and chidamide, inducing the expression of the oligodendrocyte differentiation marker (*Myelin Basic Protein*, MBP) and enhancing the antiproliferative effect of decitabine in GBM8401 and U87MG lines, without damaging normal SVGp12 cells (Liao et al. 2019).

Recently, the potential of fucoidan in counteracting the side effects of chemotherapy has been emerged.

In rats, coadministration of fucoidan and DOX improved cardiac function and serum levels of lactate dehydrogenase (LDH) and creatine kinase MB (CKMB), markers for cardiac injury (Zhang, Xue, et al. 2022). Therefore, fucoidan can be considered a chemotherapy adjuvant because it enhances the cytotoxic efficacy of conventional therapies and mitigates their side effects, thus improving the quality of life of cancer patients (Takahashi et al. 2018). The main therapeutic combinations studied with their observed effects are shown in Table 2.

**TABLE 2:** Effects of Fucoidan in associations with drugs.

Associations	Models	Effects/Actions	References
Fucoidan + Letrozole or Tamoxifen	Breast cancer	No alteration in pharmacokinetic parameters	Tocaciu et al. (2018)
Fucoidan + Chemotherapy	Colorectal cancer	Improved disease progression control	Tsai et al. (2017)
LMW Fucoidan +5‐Fluorouracil	Colorectal cancer	Synergistic action	Huang et al. (2021)
LMW Fucoidan + Oxaliplatin	Pancreatic cancer	Synergistic action	Deng et al. (2024)
LMW Fucoidan + Oligomycin	Triple‐negative breast cancer	↑ Cytotoxic effect	Zueva et al. (2023)
OF + Decitabine + Chidamide	Glioblastoma	↑ MBP, ↑ Antiproliferative effect	Liao et al. (2019)
Fucoidan + Doxorubicin	C57BL/6J mice	↑ Cardiac function, ↓ LDH and CKMB	Zhang, Xue, et al. (2022)

Abbreviations: CKMB, creatina chinasi MB; LDH, lattato deidrogenasi; LMW, low molecular weight; MBP: Myelin basic protein; OF: Oligo‐Fucoidan.

### 4.2 Drug Delivery System

In recent years, fucoidans are increasingly used as delivery systems of bioactive compounds, thanks to their ability to form nanosystems such as nanoparticles, nanoemulsions, nanocapsules and hydrogels, thus improving the stability of the active ingredients and allowing their targeted delivery in the body, a controlled release over time and an increase in bioavailability (Zhang, Wei, and Xue 2022).

Nanoparticles are formed by electrostatic interaction (essential for the effective encapsulation of active ingredients) between fucoidans and cationic polymers such as chitosan (Luo et al. 2014; Zhang, Wei, and Xue 2022). These nanoparticles are particularly sensitive to pH, making them ideal for the controlled release of encapsulated active ingredients (Huang et al. 2014; Tran et al. 2020).

Fucoidan/chitosan nanoparticles remain stable in acidic environments, but become unstable when the pH exceeds 6.5, promoting the degradation of the nanoparticle and consequently the release of the active compound (Lee and Huang 2019). A formulation with a ratio of 5:1 Fucoidan/Chitosan showed good stability at pH 3 and an effective ability to deliver methotrexate, improving its oral bioavailability (Coutinho et al. 2020). These nanosystems, biocompatible with fibroblasts and keratinocytes, also improved the skin permeation of the drug and reduced the expression of pro‐inflammatory cytokines and TNF‐α (Barbosa et al. 2019). In addition, pH‐sensitive and mucoadhesive fucoidan/chitosan nanoparticles, designed for oral administration of methotrexate, have been developed in the treatment of lung cancer to improve the oral bioavailability of the drug and the induction of apoptosis in cancer cells (Coutinho et al. 2020).

In chemotherapy, fucoidan/polyethylene cationic nanoparticles, loaded with doxorubicin, have demonstrated remarkable efficiency in delivering the drug directly to the tumor site, increasing the therapeutic efficacy of doxorubicin in the treatment of breast cancer (Pawar et al. 2019).

The fucoidan/chitosan combination also protects insulin from degradation, with a controlled release in simulated gastric and intestinal pH environments, making it more effective in the treatment of diabetes than free insulin (Tsai et al. 2019).

Another interesting formulation concerns fucoidan/protein composite nanoparticles, mainly using zein as a cationic biopolymer (Mensah et al. 2023). Zein is a hydrophobic protein that, thanks to its amphiphilic properties, is able to self‐assemble into colloidal particles, favoring the encapsulation of both lipophilic and water‐soluble compounds (Voci et al. 2020).

In recent years, it has been widely used for the development of drug delivery systems aimed at improving the bioavailability of numerous active ingredients, including anticancer drugs such as paclitaxel (Celano et al. 2022). Under simulated gastrointestinal conditions the nanosystems zein(Z)/fucoidan(F)‐based nanoparticles in different ratios (10Z:1F, 5Z:1F and 2Z:F1) in which encapsulated pterostilbene (chemically unstable antioxidant) showed high encapsulation efficiency and a more controlled release of the active substance compared to zein‐only nanosystems (Liu, Chen, et al. 2020; Liu, Qin, et al. 2022).

Similar results were also obtained with resveratrol, in which the encapsulation of fucoidan/zein nanoparticles allowed a targeted and controlled release, compared to resveratrol in free form, improving its adsorption, bioavailability, and consequently oncoprotective and anti‐aging effects (Liu, Qin, et al. 2022).

At the same time, silver nanoparticles (AgNPs) fucoidan‐coated extracted from *Turbinaria decurrens* showed important antibacterial activity, in particular on Gram‐negative bacteria ( *Pseudomonas aeruginosa* and *Escherichia coli* ) (Shanthi et al. 2021). The antibacterial action of coated AgNPs was superior to that of fucoidan alone, as they provide a higher surface‐to‐volume ratio that facilitates interaction with bacterial cells, resulting in increased antimicrobial activity (Shanthi et al. 2021).

In the ophthalmic setting, hydrogel biofilms composed of poly(2‐hydroxyethyl methacrylate) (pHEMA) and fucoidan have shown good antibacterial activity (Lee, Kim, and Cho 2012) potentially useful in eye treatments and in the production of medical devices for the eyes, where bacterial infections are a significant risk and the use of biocompatible materials is essential to avoid adverse effects (Lee, Kim, and Cho 2012).

Table 3 summarizes recent fucoidan‐based drug delivery systems, along with the observed effects and their therapeutic applications.

**TABLE 3:** Fucoidan‐based drug delivery systems.

Drug delivery system	Effects	Applications	References
Fucoidan/Chitosan nanoparticles	Release active compound at pH 6.5	Oral methotrexate delivery	Coutinho et al. (2020); Lee and Huang (2019)
Fucoidan/Chitosan nanoparticles	Improved oral bioavailability and induces apoptosis in lung cancer cells	Lung cancer treatment (oral)	Coutinho et al. (2020)
Fucoidan/Polyethyleneimine nanoparticles	Increased efficacy of Doxorubicin in breast cancer	Breast cancer chemotherapy	Pawar et al. (2019)
Fucoidan/Chitosan nanoparticles	Protection insulin degradation	Diabetes	Tsai et al. (2019)
Fucoidan/Zein nanoparticles	Efficient encapsulation and controlled release of pterostilbene	Drug Delivery	Liu, Chen, et al. (2020); Liu, Qin, et al. (2022)
Fucoidan/Zein nanoparticles	Controlled release and improved bioavailability of Resveratrol	Anti‐aging and oncoprotection	Liu, Qin, et al. (2022)
Fucoidan‐coated silver nanoparticles (AgNPs)	Enhanced antibacterial activity	Antibacterial agents	Shanthi et al. (2021)
Fucoidan/pHEMA hydrogel films	Antibacterial activity	Ophthalmic devices and treatments	Lee, Kim, and Cho (2012)

Marine polysaccharides, thanks to their nontoxicity and biocompatibility, have proven to be excellent candidates for biomedical applications, both as vectors of imaging markers and in tissue regeneration processes (Chollet et al. 2016; Lee et al. 2017).

LMW fucoidan promoted the proliferation of human osteoblasts, increasing alkaline phosphatase activity and type I collagen expression, improving mineralization and subsequently promoting bone tissue regeneration (Changotade et al. 2008).

In a study conducted by Han et al. (2015) fucoidan increased the survival of mesenchymal stem cells by inhibiting reactive oxygen species, protecting them from ischemia‐induced apoptosis (Han et al. 2015).

The formulation of chitosan/alginate/fucoidan (CAFFS) scaffolds improved the cytocompatibility, proliferation, alkaline phosphatase secretion, and mineralization of MG63 osteosarcoma cells, compared to chitosan/alginate (CAS) scaffolds alone (Jeong et al. 2013; Lowe et al. 2016; Venkatesan et al. 2014).

In a further study, a tricalcium phosphate‐chitosan‐fucoidan biocomposite was formulated, the addition of fucoidan to which increased osteocalcin release, enabling the osteogenic differentiation of human mesenchymal stromal cells in vitro (Puvaneswary et al. 2015).

Furthermore, polycaprolactone (PCL)/fucoidan composite scaffolds for bone tissue regeneration allowed an increase in the hydrophilic properties of the nanosystem and a better cellular adhesion to the surface three times greater than the PCL scaffold, also determining an increase in biocompatibility on osteoblast‐like cells (MG63) (Jin and Kim 2011; Lee, Jin, et al. 2012). Table 4 summarizes recent fucoidan‐based bioformulations and describes their effects on tissue regeneration processes.

**TABLE 4:** Fucoidan‐based bio‐formulations and applications.

Bio‐formulation	Effects	Applications	References
LMW fucoidan	↑ Proliferation of human osteoblasts ↑ Alkaline phosphatase activity ↑ Type I collagen expression and mineralization	Bone tissue regeneration	Changotade et al. (2008)
Chitosan/alginate/fucoidan (CAFFS)	↑ Cytocompatibility, proliferation, alkaline phosphatase secretion, and mineralization of MG63	Osteosarcoma	Jeong et al. (2013); Lowe et al. (2016); Venkatesan et al. (2014)
Tricalcium phosphate‐chitosan‐fucoidan	↑ Osteocalcin release ↑ Osteogenic differentiation	Bone tissue regeneration	Puvaneswary et al. (2015)
Polycaprolactone (PCL)/fucoidan	↑ Cellular adhesion	Bone tissue regeneration	Jin and Kim (2011); Lee, Jin, et al. (2012)

## 5 Conclusions

Over the past decade, the demand for natural formulations has increased rapidly, and the exploration of bioactive molecules derived from natural sources that can be used for these purposes is increasingly growing.

Particular attention has been paid to fucoidan, a sulfated polysaccharide derived from marine algae with multiple documented biological properties influenced by structural parameters such as molecular weight, concentration, degree of sulfation, and purity.

Fucoidan is a biocompatible and biodegradable polymer and, thanks to its antitumor, anti‐inflammatory, antioxidant, immunomodulatory, anticoagulant, antiviral, neuroprotective, and prebiotic effects, is a promising molecule for a wide range of applications.

In this review, we summarize and discuss its biological effects, including reduction of oxidative stress and activity on pro‐inflammatory cytokines, modulation of apoptotic processes, increased expression of Beclin‐1 and LC3‐II, and induction of signaling pathways such as JAK2‐STAT3.

Furthermore, we highlight its role in modulating autophagy and epigenetic mechanisms through direct action on DNA, further highlighting the pretreatment of exosome‐derived mesenchymal stem cells, in which the presence of miRNAs (miR‐146b‐5p, miR‐23b‐5p, and miR‐615) also contributed significantly to the anti‐inflammatory effects.

From a pharmacokinetic perspective, low bioavailability and rapid elimination remain significant limitations. Nanoformulations, such as nanoparticle encapsulation, offer promising prospects for improving stability and targeted release.

Recently, Fucoidan has also emerged as a component in drug delivery systems, reducing side effects and potentially improving the efficacy of encapsulated active ingredients.

For this reason, it also offers the possibility of being used as an adjuvant in chemotherapy to improve the clinical efficacy of chemotherapeutics, reduce drug resistance, and consequently improve patients' lives.

However, despite its benefits for human health and potential nutraceutical, pharmaceutical, cosmetic, and food applications, critical issues remain related to its complex chemical and physical characterization, including that of the algal species of origin, and the need to standardize production processes to ensure quality and quantity. Therefore, although research on the toxicity of fucoidan is still limited, and despite the numerous documented beneficial effects and numerous in vitro and in vivo studies that have not revealed significant adverse effects, further research is needed to understand its pharmacokinetic and pharmacodynamic profile to ensure its safe and effective use. Future clinical directions should focus on its potential as an adjuvant in chemotherapy regimens and its use as a therapeutic drug delivery system. Its potential as a scaffold and/or functional polymer, both fundamental to tissue engineering and regenerative medicine, should be further explored.

## Author Contributions

**Marilena Celano:** conceptualization, writing – review and editing, supervision, writing – original draft. **Sharon Critelli:** conceptualization, writing – original draft.

## Funding

This work was supported by Università degli Studi Magna Graecia di Catanzaro; Dipartimento di Scienze della Salute.

## Conflicts of Interest

The authors declare no conflicts of interest.

## Data Availability

Data sharing not applicable to this article as no datasets were generated or analyzed during the current study.
